# Unmet Needs and Perspectives in Oral Cancer Prevention

**DOI:** 10.3390/cancers14071815

**Published:** 2022-04-02

**Authors:** Jebrane Bouaoud, Paolo Bossi, Moshe Elkabets, Sandra Schmitz, Léon C. van Kempen, Pierre Martinez, Sankar Jagadeeshan, Ingrid Breuskin, Gerwin J. Puppels, Caroline Hoffmann, Keith D. Hunter, Christian Simon, Jean-Pascal Machiels, Vincent Grégoire, Chloé Bertolus, Ruud H. Brakenhoff, Senada Koljenović, Pierre Saintigny

**Affiliations:** 1 Centre de Recherche en Cancérologie de Lyon, Centre Léon Bérard, CNRS 5286, INSERM 1052, Université Claude Bernard Lyon 1, University Lyon, F-69008 Lyon, France; pierre.martinez@lyon.unicancer.fr; 2 Department of Translational Research and Innovation, Centre Léon Bérard, Université Claude Bernard Lyon 1, University Lyon, F-69008 Lyon, France; vincent.gregoire@lyon.unicancer.fr (V.G.); chloe.bertolus@aphp.fr (C.B.); 3 Department of Maxillo-Facial Surgery, Assistance Publique des Hôpitaux de Paris, Sorbonne Université, Hôpital Pitié-Salpêtrière, F-75013 Paris, France; 4 Medical Oncology, ASST Spedali Civili Brescia, I-25064 Brescia, Italy; paolo.bossi@unibs.it; 5 Department of Medical and Surgical Specialties, Radiological Sciences and Public Health, University of Brescia, I-25123 Brescia, Italy; 6 The Shraga Segal Department of Microbiology, Immunology and Genetics, Ben-Gurion University of the Negev, Beer-Sheva 8410501, Israel; moshee@bgu.ac.il (M.E.); jagadees@post.bgu.ac.il (S.J.); 7 Faculty of Health Sciences, Ben-Gurion University of the Negev, Beer-Sheva 8410501, Israel; 8 Department of Medical Oncology and Head and Neck Surgery, Institut Roi Albert II, Cliniques Universitaires Saint-Luc and Institut de Recherche Clinique et Expérimentale (Pole MIRO), UCLouvain, 1200 Brussels, Belgium; sandra.schmitz@uclouvain.be (S.S.); jean-pascal.machiels@saintluc.uclouvain.be (J.-P.M.); 9 Department of Pathology and Medical Biology, University Medical Center Groningen, University of Groningen, 9712 CP Groningen, The Netherlands; l.van.kempen@umcg.nl; 10 Department of Head and Neck Oncology, Gustave Roussy Cancer Campus, F-94805 Villejuif, France; ingrid.breuskin@gustaveroussy.fr; 11 Department of Dermatology, Erasmus MC, University Medical Center Rotterdam, Room Ee-1691, P.O. Box 2040, 3000 CA Rotterdam, The Netherlands; gpuppels@riverd.com; 12 INSERM U932 Research Unit, Department of Surgery, Institut Curie, PSL Research University, F-75006 Paris, France; caroline.hoffmann@curie.fr; 13 Unit of Oral and Maxillofacial Pathology, School of Clinical Dentistry, University of Sheffield, Sheffield S10 2TA, UK; k.hunter@sheffield.ac.uk; 14 Department of Otolaryngology and Head and Neck Surgery, Lausanne University Hospital, 1011 Lausanne, Switzerland; christian.simon@chuv.ch; 15 Radiation Oncology Department, Centre Léon Bérard, Université Claude Bernard Lyon 1, University Lyon, F-69008 Lyon, France; 16 Cancer Center Amsterdam, Section Head and Neck Cancer Biology & Immunology, Otolaryngology and Head and Neck Surgery, Vrije Universiteit Amsterdam, Amsterdam UMC, 1081 HV Amsterdam, The Netherlands; rh.brakenhoff@amsterdamumc.nl; 17 Department of Pathology, Erasmus MC, University Medical Center Rotterdam, 3015 GD Rotterdam, The Netherlands; senada.koljenovic@uza.be; 18 Department of Medical Oncology, Centre Léon Bérard, Université Claude Bernard Lyon 1, University Lyon, 28 Promenade Léa et Napoléon Bullukian, F-69008 Lyon, France

**Keywords:** oral potentially malignant disorders, oral preneoplasia, oral cancer, prevention, diagnosis

## Abstract

**Simple Summary:**

Oral cavity is the most common site of head and neck cancer which is ranked as the eighth most common cancer worldwide. Oral cancer treatment is often associated with significant morbidity and is sometimes ineffective. These cancers, mainly due to tobacco and alcohol consumption, can develop from oral potentially malignant disorders, the most common of which is oral leukoplakia. Some of these oral potentially malignant disorders disappear, while others will transform to oral cancer. Patients may also develop cancer in the field of cancerization. Unfortunately, except for the surgical excision of lesions with dysplasia, there is no effective intervention to effectively prevent transformation or cancer development in the field of cancerization. Moreover, no standardized biomarker has been clearly identified as sufficient to predict malignant transformation. In this article, several experts discuss the main challenges in oral cancer prevention, in particular the need (i) to define new a new classification system integrating cellular and molecular features aiming (ii) at better identifying patients at high risk of malignant transformation, and (iii) at developing treatment strategies to prevent their malignant transformation of oral potentially malignant disorders.

**Abstract:**

Oral potentially malignant disorders (OPMD) may precede oral squamous cell carcinoma (OSCC). Reported rates of malignant transformation of OPMD range from 3 to 50%. While some clinical, histological, and molecular factors have been associated with a high-risk OPMD, they are, to date, insufficiently accurate for treatment decision-making. Moreover, this range highlights differences in the clinical definition of OPMD, variation in follow-up periods, and molecular and biological heterogeneity of OPMD. Finally, while treatment of OPMD may improve outcome, standard therapy has been shown to be ineffective to prevent OSCC development in patients with OPMD. In this perspective paper, several experts discuss the main challenges in oral cancer prevention, in particular the need to (i) to define an OPMD classification system by integrating new pathological and molecular characteristics, aiming (ii) to better identify OPMD at high risk of malignant transformation, and (iii) to develop treatment strategies to eradicate OPMD or prevent malignant transformation.

## 1. Introduction

Oral cavity is the most common site of Head and Neck Squamous Cell Carcinoma (HNSCC), which is ranked as the eight most common cancer worldwide [1]. Oral SCC (OSCC) is a major cause of morbidity and mortality [2,3]. OSCC are preceded by mucosal precancerous changes that might be visible as white (leukoplakia) or red (erythroplakia) lesions, but are mostly not macroscopically visible, which explains that most OSCC seem to develop *de novo*. However, the preceding precancerous changes can present under the microscope as abnormal mucosal epithelium, also indicated as dysplasia, graded as mild moderate and severe, or they can be identified by genetic markers. In 2017, the World Health Organization (WHO) defined oral potentially malignant disorders (OPMD) as “clinical presentations that carry a risk of cancer development in the oral cavity, whether in a clinically definable precursor lesion or in clinically normal mucosa” [4]. Thus, OPMD may precede OSCC, and may be visible or not [5]. While it is traditionally assumed that OPMD and OSCC are associated with similar risk factors (e.g., alcohol, tobacco, betel quid), a proportion of OPMD and OSCC cases occur in the complete absence of any identifiable risk factor, particularly in young patients who have never been drinkers or smokers [6,7,8,9]. The overall worldwide prevalence of OPMD is about 4.5% [10]. The main risk factors of malignant transformation of OPMD described to date are patient-related, clinical (e.g., female, >50 years; non-smoker with a nonhomogeneous red lesion of the tongue and floor of mouth >200 mm^2^ and existing for several years; history of previous OSCC; diabetes mellitus), tumor-related, histological (i.e., severe dysplasia), and molecular factors (i.e., aneuploidy, loss of heterozygosity [LOH]). The reported malignant transformation rates range from 3 to 66%, indicating that variable definitions may be used, data with different follow-up periods have been collected and the existence of histological and, in particular, molecular heterogeneity of OPMD [11,12,13,14,15]. For OPMDs that are visible, standard policy is to take multiple, repeated, and deep incision biopsies to check for invasive growth and dysplasia. Treatment of the OPMD may prevent malignant transformation and improve outcome [6,11]. The surgical excision of OPMD can decrease the risk of malignant transformation at the same site, but it does not eliminate the risk of subsequent development of SCC at other sites [16]. To date, no standard therapy has been shown to be effective in patients with OPMD to prevent OSCC development in the entire field of cancerization [17].

The main challenges are (i) to define an OPMD classification system integrating new pathological and molecular characteristics, aiming (ii) to better identify OPMD at high risk of malignant transformation, and (iii) to develop prevention strategies that would treat both the visible lesion and the entire field of cancerization [18,19]. Large longitudinal studies of OPMD cases with malignant transformation, as the most relevant clinical outcome, are required.

## 2. Pathological Perspective

As defined in the recent OPMD WHO classification, OPMD include fifteen disorders affecting the oral mucosa (e.g., leukoplakia, erythroplakia, proliferative verrucous leukoplakia, oral submucous fibrosis…) and which are either secondary to genetic aberrations, exposure to exogenous factors such as tobacco and/or immune-mediated disorders or related to rare inherited diseases [4,20]. The different histologic features, especially those usually used to grade dysplasia (architectural and cytologic changes…) have been reviewed elsewhere [21].

The histopathological diagnosis and grading of dysplasia are the gold standard in guiding OPMD management. Unfortunately, especially in the oral cavity, it is challenging due to the high degree of inter and intra-observer variability, resulting in limited value of grading of dysplasia as a predictive factor for OPMD malignant transformation [22,23]. The WHO classification postulates that the more advanced the degree of dysplasia, the higher the likelihood of developing oral squamous cell carcinoma (OSCC). However, the literature reports that OSCC may also arise from seemingly non-dysplastic epithelium. The histology of these lesions is subtle and easily underdiagnosed. In particular, by studying the abnormalities in the mucosa surrounding OSCC, it was recently shown that the dysplastic changes are most commonly subtle (70%, with the features of so-called differentiated dysplasia) and therefore may easily be undervalued by the pathologist [24]. To improve the dysplasia diagnosis, authors proposed refined histopathological criteria, and have shown that immunohistochemistry with antibodies against cytokeratin 13, cytokeratin 17, and Ki67 is a useful diagnostic adjunct. It has been shown that, compared to the classic histologic criteria (Who 2017), differentiated dysplasia improves the prediction of oral leukoplakia at increased risk of malignant progression [25]. To address the issues in histological diagnosis and grading of dysplasia, we should develop refined and standardized histopathological criteria encompassing the various histological appearances for reliable diagnosis of OPMD and implement validated immunohistochemical and molecular biomarkers.

In addition, Artificial Intelligence methods are becoming a powerful diagnostic adjunct [26]. In particular, machine learning and deep learning algorithms are promising for diagnostic support (enhance laboratory efficiency and quality assurance), as disruptive technology to standard biomarkers, and to derive patterns not achievable by a human observer [27]. Although this field is rapidly evolving, currently very few algorithms have reached clinical implementation [28].

## 3. Biomarkers, Prospective High-Risk Cohorts with Embedded Trials

Besides the clinical and histological characteristics of OPMD [4], several biomarkers have been proposed to identify patients with OPMD at high risk of OSCC development [29]. LOH at specific chromosomal sites (3p14 or 9p21) has been validated prospectively [30]. LOH was also found to be a biomarker predicting the development of second oral malignancies in patients with an OPMD, subsequent to the treatment of a OSCC [31,32]. Prospective cohorts with long-term follow-up of patients with OPMD are needed to identify other predictive biomarkers that may be used for clinical practice.

## 4. Biology of Precancerous Changes

In 1953, Slaughter et al., concluded from histopathological studies of oral cancer specimen: “From the foregoing observations it would appear that epidermoid carcinoma of the oral stratified squamous epithelium originates in a process of “field cancerization,” in which an area of epithelium has been preconditioned by an as-yet-unknown carcinogenic agent. Such a carcinogenic influence if operative enough in time and intense enough in exposure produces an irreversible change in cells and cell groups in the given area, so that change of the process toward cancer becomes inevitable” [33]. It is remarkable that this model was already reported before tobacco and alcohol were identified as the major culprits of OSCC, and before the scientific world had any clue on molecular carcinogenesis and the role of mutated cancer genes. At present, we know that cancer arises by the accumulation of genetic and epigenetic changes, causing a changed circuitry of many signal transduction routes and invoking the acquired capabilities of cancer cells characterized as the “hallmarks of cancer” [34]. Hence, the onset and driving force of carcinogenesis is the accumulation of genetic changes, albeit stroma interactions likely play a role in parallel. The genetic changes occurring during oral carcinogenesis are now well defined [33,35,36,37,38]. Typical chromosomal changes such as loss of 3p, 9p, and 17p that are frequently found in invasive HNSCC are also found in precancerous changes and are in fact the most accurate predictors of malignant transformation of the OPMD, as discussed above [30].

Given the causal role of genetic changes in carcinogenesis, the upper aerodigestive tract field cancerization may be explained, at least partially, by the accumulation of genetic changes in the mucosal keratinocytes. There are no specific markers of stem cells in the mucosa, but we may assume that these exist in the basal layer of the mucosal epithelium. The stemness of such cells is not intrinsic and fixed, but most likely the result of a dynamic process as it is in the intestine [39]. These stem cells form the basis of the mucosal units of transit, amplifying cells and differentiating cells in areas of approximately 200 cells wide, which together make up the mucosal epithelium. This clonal unit was demonstrated in mouse epidermis using Axin2 lineage tracing experiments [40]. A somatic mutation in such a cell with stemness properties will give rise to a mutated clonal unit as first described in 2002 using *TP53* mutations as a molecular marker [41]. These rare somatic mutations in cells have since been shown in numerous tissues and are studied using next generation sequencing approaches [42,43]. The mutated cells compete with the wild type cells. In the skin, UV-induced cell death of normal cells supports the extension of the preneoplastic cells [44]. In the oesophagus, oxidative stress has been identified as a potential factor that supports the proliferation of TP53-mutated cells over the wild type cells [45]. When applying N-acetylcysteine (NAC) as oxidative stress reducing agent, the balance was shifted in advantage of wild type cells. However, no effect of NAC to prevent recurrent cancer or second primary tumours in both lung and head and neck cancer patients was seen in the EUROSCAN trial [46].

Besides environmental factors that may favour the growth of genetically damaged cells, the accumulation of subsequent genetic alterations may induce a growth advantage and change the balance between normal cells and genetically damaged cells, the latter displacing the normal mucosa by so far unresolved mechanisms. It is likely not related to proliferation rate as normal keratinocytes, precancer and cancer cells may have comparable cell division times, at least *in vitro* [47].

## 5. Field of Cancerization

A field should be defined as a group of cells with tumour-associated somatic genetic alterations. Irrespective of the underlying biology and cellular interaction, the preneoplastic fields will develop in time and can reach dimensions greater than 10 cm in diameter. As explained above, the minority is clinically visible as an asymptomatic persistent white or red lesion that cannot be rubbed off [20]. The clinical aspect is poorly specific of OPMD, given that not all lesions harbour histologically proven dysplasia [25]. Hence, the visible lesions form the tip of the iceberg. Indeed, some normal surgical margins of oral cancer specimen showed genetic changes, indicating that not all precancerous fields are recognized by histology, and that we must rely on genetic markers to identify all potentially malignant fields. However, with the introduction of differentiated dysplasia as novel morphological entity [24,25], this may change soon. Whether they are visible or not, these potentially malignant changes may transform into invasive cancers. The tumours are diagnosed and treated, but particularly when these fields are not visible to the naked eye, they may stay behind and cause local relapses clinically diagnosed either as local recurrence or second primary tumour, depending on the distance (2 cm and/or different subsite) and the time interval (3 years) [35,36]).

*In vitro* cultures of visible lesions were reported in 2002 [48]. More recently, 98 2D cultures from normal appearing mucosa of the surgical margins of patients with primary HNSCC were generated and characterized for their molecular alterations and the number of population doublings (PDs) [47]. Cultures with more than 20 PDs and a random selection of nine other cultures with a normal life span (<20 PDs) were analysed for copy number changes and for mutations of the ten key HNSCC driver genes using target-enrichment sequencing. Irrespective of the lifespan of < or >20 PD, in 50% of the cultures somatic genetic changes were identified with a large variety in type and number. Despite many genetic alterations in some cultures and an apparent immortal lifespan, none formed tumours in immunodeficient mice, demonstrating the lack of invasive capacity and confirming the precancerous state [48]. This supports that acquisition of immortality is an earlier event during OSCC progression than acquisition of invasive properties. Most frequently mutated genes were *TP53*, *NOTCH1* and *FAT1*, whereas *CDKN2A* showed frequent copy number losses. Most intriguingly, in four cultures copy number changes were found but no mutations in key driver genes, suggesting that carcinogenesis may start with copy number changes, although such precancerous cells may never transform.

In summary, field cancerization has been well characterized in genetic terms, the cells can be cultured and even used for therapeutic target screening [49,50]. A field should be defined as a group of cells with tumour-associated somatic genetic alterations. A field should be larger than the clonal unit and, consequently, larger than at least 200 cells wide and can reach dimensions of up to 10 cm in diameter. Some fields present as dysplasia under the microscope, and some are macroscopically visible as a non-specific persistent white or red lesion. These fields contain a variety of genetic changes, but typically also mutations in the cancer driver genes of head and neck cancer. They develop by a process of somatic mutation in relation to aging and carcinogen exposure. The reason as to why the normal epithelium is displaced remains an enigma. Enhanced proliferation seems logical but is likely not the cause, and biological processes perhaps stimulated by environmental cues may be more likely.

## 6. The OPMD Immune Microenvironment (IME)

The interplay between OPMD and IME has been poorly explored, while it appears as a promising and actionable target [51,52]. Briefly, compared to OPMD that transformed into OSCC, patients with dysplastic OPMD and no subsequent malignant transformation had significantly more infiltrating CD3+, CD4+ and CD8+ T-cells and decreased T-regulatory cells [53,54,55,56]. Furthermore, the progression from OPMD to OSCC has shown increased number of CD163+ cells (M2 Macrophages), PD-L1 expression and a decreased number of CD8+ cells [52,53,56,57,58,59]. More recently, the Saintigny Team (JB, PS) studied the dynamic of the IME in the 4-NQO murine model of oral carcinogenesis [60], an accepted model for the human disease in particular at early steps of tumorigenesis [61]. They found that changes in the composition of immune infiltrate (T-cells, B-cells, M1/M2 macrophages) can already be observed in histologically proven premalignant stages. Transcriptomic changes revealed activation of immune related processes at early steps of oral carcinogenesis. On the other hand, when the gene expression data of 86 patients with OPMD were challenged with transcriptomic features coming from HNSCC patients, the lesions could be stratified in several clusters, and the OPMD from the mesenchymal, hypoxia and classical molecular subgroups showed a higher risk of malignant transformation in comparison with the immune-related ones [62].

It is tempting to speculate on OPMD within the concept of “immunoediting”, hypothesizing that these lesions are in the equilibrium phase of a dynamic process between the malignant transformation and surveillance of the immune system. One hypothesis is that malignancy will develop in the presence of an immunosuppressive microenvironment. Another hypothesis is that OPMD do not elicit a sufficient immune response, and that for two main reasons: (i) OPMD highly resemble “self” and are not detected as non-self by the immune system; (ii) OPMD barely induce local tissue-damage and therefore insufficiently release the immune-attracting damage-associated molecular patterns.

Overall, while promising, our knowledge of the complex and dynamic nature of the OPMD IME remains incomplete, which might explain the failure of immunoprevention strategies [63,64]. Thus, further characterization of the dynamic changes in immune response during oral carcinogenesis is required [51,52], especially differences between OPMD that subsequently transformed into OSCC and those that did not.

## 7. Oral Microbiome

The study of the potential contribution of the microbiome in the carcinogenesis of different cancer types including OSCC is emerging [65]. Regarding the very few studies which have reported the microbiome composition associated with OPMD, results are heterogeneous and difficult to compare because of diversity in microbiota and methodological heterogeneity [66,67]. Briefly, it was suggested that the microbiota may contribute to tumorigenesis, both directly (production of microbial genotoxin inflicting DNA damages), and indirectly through its interplay with the immune system (stimulation of chronic inflammation alters the immune responses and aberrant immune responses facilitate dysbiosis, especially in aging context) [68]. Moreover, the dysregulation by the microbiome of some physiological activities that are critical for oral carcinogenesis (nitrogen transport, response to stress, interspecies interactions, Wnt pathway modulation, and amino acid and lipid biosynthesis) were identified using the 4-NQO mice model [69]. Overall, the understanding of the role of the oral microbiome in carcinogenesis is still an area of investigation [67].

## 8. Early Diagnosis of OPMD

The early detection of OPMD serves the purpose of secondary prevention of oral cancer [70]. Examination of the oral cavity (visual inspection and palpation) is the conventional method for identifying and monitoring OPMD. However, clinical recognition of OPMD is challenging [5]. Thus, methods to enhance the early detection of OPMD are required [4,5,71].

In 2008, the International Agency for Research in Cancer (IARC) published a digital manual to help physicians in this aim. Furthermore, non-invasive *in vivo* optical imaging provides unique opportunities for real-time diagnosis of oral pre-malignancies. These techniques are mainly autofluorescence imaging (AFI), targeted fluorescence imaging (TFI), high-resolution microendoscopy (HRME), narrow band imaging (NBI) and Raman spectroscopy (RS) (Table 1) [72,73].

Using AFI, altered and dysplastic tissues appear darker compared to the healthy surrounding tissue (autofluorescence loss). AFI devices displayed superior accuracy levels in the identification of OPMD compared to clinical examination [74]. AFI devices evaluated for early diagnosis of OPMD are practical and cost-effective but suffer from low specificity [5]. Moreover, mucosa with hyperkeratinisation such as some oral leukoplakia can demonstrate increased autofluorescence when compared to normal mucosa, which limits the ability to detect malignant change within such lesions [75]. TFI utilizes a targeting fluorescence probe which can specifically target some elements by approved antibodies (targeted immune-fluorescence imaging). However, the lesion heterogeneity could decrease the TFI sensitivity.

NBI visualizes the angiogenic patterns within and surrounding lesions. NBI as an endoscopic system is widely available and easy to use [5,76,77]. Moreover, the neoangiogenesis-related morphological changes, especially the abnormal intraepithelial capillary loops (ICPL) patterns, have been widely reported [5,75]. Unfortunately, IPCL patterns characterization is subjective and the visualization of microvessel architecture may be affected by various factors. Artificial intelligence may make the prediction of malignant transformation more objective and with greater accuracy [26].

HRME is cost effective, non-invasive and provides real-time high-resolution microscopic images (in situ “optical biopsy”) [78]. HRME has demonstrated high sensitivity and specificity. However, HRME is not commercially available, its contrast agent is not yet approved, and the field of view is limited [5].

RS is a non-destructive vibrational spectroscopic technique [79,80,81]. Raman spectra represent the overall molecular composition of the tissue and can be used to distinguish healthy tissue from (pre-)malignant tissue [5]. RS is a promising tool for early diagnosis/biopsy guidance and follow up (optical biopsy) of OPMD but required further development [82].

Other imaging techniques to detect OPMD are optical coherence tomography, elastic scattering spectroscopy, diffuse reflectance spectroscopy, confocal laser endomicroscopy and confocal reflectance microscopy, but they are not widely developed [5,83]. Vital staining (toluidine blue, Methylene blue, Rose Bengal and Ludo’s iodine) are sensitive, simple, rapid, efficient and low-cost techniques [5,77,84] but false positive results are frequent, and their application is not without issues.

In summary, the previously described techniques are promising with high sensitivity to detect OPMD but suffer from poor specificity. This is not only due to inherent limitations of the techniques, but also to the lack of a good histological gold standard, which renders the development of predictive algorithms based on optical methods very difficult [5,75,84]. To overcome the technical part of the problem, a combination of techniques, e.g., combining AFI and HRME, are interesting [85,86]. Further investigations (large randomized clinical trial with long follow-up) are needed.

## 9. Preclinical Models

### 9.1. In Vitro Tissue Culture Models

#### 9.1.1. 2D Culture of Cell Lines

There are many reports of cell lines being established from OPMD biopsies (Table 2). These OPMD cell line model systems recapitulate the key characteristics of the clinical lesions closely and have been used to study the early stages of oral cancer and malignant transformation of oral keratinocytes *in vitro* [87,88,89,90,91,92,93,94]. However, the major limitation of cell line models is that these cells fail to grow *in vivo*, thereby prohibiting the study of the involvement of the oral microenvironments.

#### 9.1.2. 3D Culture of Organotypic Co-Culture

In this method, keratinocytes are cultured at an air-to-liquid interface on a fibroblast-containing collagen type I matrix. While several refinements have been proposed to overcome the major limitations of the classically used collagen-based connective tissue equivalent (deficit of complex structural heterogeneity and collagen fibre crosslinking present in mature connective tissue, induction of artificial epithelial invasion by lose of biostability over a long period of culture and lack of a well-defined continuous basement membrane between the epithelium and connective tissue) [95], to date, most organoids lacked vasculature, fibroblasts and immune cell components that are known to influence malignant transformation, which make them not a true representation of *in vivo* transformation of OPMD to OSCC.

Recently, to mimic the oral mucosal complexity, progress has been achieved in designing more complex tissue engineering techniques in organotypic co-cultures that includes the incorporation of blood capillaries to the cell surface [96], culturing oral keratinocytes with fibroblasts [97], immune cells [98], and oral microbiota [99,100]. As protocols and analysis methods continue to improve, these 3D techniques will become more accessible within the said field.

#### 9.1.3. *In Vivo* Rodent Models

1. Carcinogen-induced models

Several agents, including coal tar, cigarette smoke, benzo[a]pyrene (B[a]P), 3-methylcholanthrene, 7,12-dimethylbenz(a)anthracene (DMBA) and 4-nitroquinoline-1-oxide (4-NQO) have been used to induce OSCC in rodent models. In particular, the 4-NQO-induced oral carcinogenesis murine model closely resembles human OSCC in terms of pathogenesis, pathological changes, host immune activity, and molecular levels, thus making this model widely acceptable to study OSCC, especially for the identification of biomarkers for early diagnosis and the transformation of the epithelium [61]. The major limitations of the carcinogen-induced models are (i) the requirement of prolonged animal and carcinogen handling, making them laborious and time-consuming, (ii) the resulting tumours do not recapitulate the tumours in patients, and (iii) it is not possible to study specific gene alterations in the development and malignant transformation process.

2. Genetically engineered mouse models (GEMMs)

GEMMs that allow oncogene activation and/or tumour suppressor inactivation solely in stratified epithelia of the oral cavity under the control of inducible promoters are extensively used to study OPMD [101]. While promising, there are still several barriers to their full application in understanding the OPMD malignant transformation. The main limitation is that these models do not reflect human oral pathogenesis in terms of the degree of gene expression during the transformation process. Secondly, these models have low specificity to form premalignant lesions by gene activation or inactivation and appear in sites other than the oral cavity. Thirdly, the introduction of exogenous genes or the knockout of endogenous genes in GEMM will occur in almost every cell which does not recapitulate the normal oral microenvironment of OPMD. Lastly, the potentially induced changes or disruptions to the oral microbiome limit the use of GEMMs for understanding the relationship between the oral microbiome and OPMD.

## 10. Prevention Strategies

### 10.1. Current Clinical Management of OPMD

To date, there has been a general consensus for the most appropriate management of OPMD [75]. Primary prevention remains the first management measure. In all cases, tobacco and alcohol consumption cessation is required to limit the risk of malignant progression, as well as the screening of whole upper aero-digestive tract mucosa for OPMD [20]. Furthermore, the histological assessment of the biopsy, especially the grading of dysplasia, should be performed both at baseline and in case of clinical modifications (macroscopic, clinic) because of its high prognostic value [12]. Surgical resection is applied when possible and certainly indicated for OPMD with moderate or severe dysplasia [20]. When surgery is not feasible (patient not operable or surgery excessively mutilating), the two available options are either destruction of the lesion (cryosurgery, carbon dioxide laser, photodynamic therapy) and/or the close surveillance with repeated biopsies. Finally, a recent Cochrane database review indicated no useful medical treatments to prevent OPMD malignant transformation [17].

### 10.2. Systemic Strategies to Prevent Malignant Transformation of OPMD

Treatment of the lesion and prevention of malignant transformation of OPMD may improve patient outcome [11]. Hence, inhibitors that eradicate the lesion, or chemopreventive agents that prevent the malignant transformation of OPMD must be developed. Several systemic agents have been tested such as bleomycin, Vitamin E, retinoids, beta carotene, lycopene and mixtures of tea [31,75,102]. However, these agents showed limited benefits. Although they caused macroscopic regression of OPMD, recurrences occurred frequently after discontinuation of treatments, and they were not shown to prevent OPMD malignant transformation [11,17].

It has been proposed to leverage premalignant biology for precision-based and more specifically immune-based cancer prevention [103,104]. Unfortunately, targeted therapies have failed to prevent malignant transformation of OPMD [31]. On the other hand, the IME is an attractive therapeutic target [51,52]. The development of multimodal immune-prevention strategies to halt OSCC progression, including immune check point inhibitors, vaccines, adjuvants activating the innate immune system and in combination with some chemopreventive agents that impact positively the tumour IME, is an interesting option [105]. In recent clinical trials evaluating PD-1- and PD-L1 targeting monoclonal antibodies (pembrolizumab and avelumab) patients with OPMD at high-risk of oral cancer development based on LOH status have been enrolled (NCT02882282 and NCT04504552), but the results are still awaited.

## 11. Conclusions and Discussion

Given the knowledge gaps in OPMD clinical management, classification, and risk stratification, as well as the lack of standardized procedures for biospecimen collection (i.e., mucosal biopsy; oral brushes; saliva), the lack of efficient, acceptable, and approved interventions to treat the whole cancerization field and the lack of a network of cooperating centres for clinical research in this area, several European experts in the field give their opinions and perspectives.

Joint efforts of academic teams and societies, clinical cancer research organizations, biotechs and pharmaceutical companies should be engaged to decipher the full temporal spectrum of the disease that may evolve to OSCC. There is need to define standardized procedures for sample collection, to refine OPMD classification and improve patients’ stratification. A biologically-driven classification of OPMD may identify clusters with actionable biology, allowing the development of prevention strategies that treat the entire field of cancerization.

There is a critical need for standardized protocols for the clinical screening and diagnosis of OPMD, in particular to encourage systematic biopsies, and for patient follow-up and treatment. Minimally invasive technologies for OPMD detection should be prioritized. For pathological diagnosis, the current gold standard, we should (i) develop standardized histopathological criteria encompassing the various histological appearances for reliable diagnosis of OPMD; (ii) implement validated immunohistochemical and molecular biomarkers; (iii) incorporate artificial intelligence for diagnostic support; and (iv) develop and implement objective detection techniques as well as non-invasive alternatives to biopsies (buccal brushes, saliva, buccal rinses, optical techniques) [83,103,104,105,106,107,108,109,110].

Prospective population-wide studies of longitudinal disease trajectories to interrogate the general medical histories of patients with cancer represent a recently developed concept to improve healthcare monitoring and reduce costs. Analysis of national or regional data hubs (e.g., clinical data warehouses, cancer registries, social security databases, hospital electronic medical records etc.) may identify disease associations occurring prior to OSCC diagnosis.

Electronic health (eHealth) interventions and patient-reported outcome tools (PROMs) dedicated to patients with OPMD to monitor disease progression, to identify early signs of transformation and to monitor the lifestyle and psychological impact of being at risk (uncertainty, anxiety and depression) [111] should be developed and evaluated. This may spare unnecessary visits and exams, while providing the best possible care.

Finally, there is a need to evaluate the socio-economic impact of preventive medicine and to perform generalizable health technology assessment; a network of centres gathering cost- and patient-related data should be built. Eventually, the aim here would be to decrease the economic burden of OSCC.

## Figures and Tables

**Table 1:** Main *in vivo* optical imaging methods that could be used as an adjunct to conventional oral examination in oral premalignant disorders screening are autofluorescence imaging (AFI), targeted fluorescence imaging (TFI), high-resolution microendoscopy (HRME), narrow band imaging (NBI), Raman spectroscopy (RS). For each method, basic principles, advantages and inconvenient are described as well as references.

*In Vivo* Optical Imaging Methods to Detect OPMD	Basic Principle	Advantages	Inconvenient	Interesting Studies on Methods to Detect OPMD
Autofluorescence imaging (AFI)	Visualization of the autofluorescence from endogenous fluorophores (NADH and FAD) Metabolic and morphologic changes related to carcinologic process led to autofluorescence loss. Altered/dysplastic mucosa appears darker compared with the healthy surroundings.	Practical Cost-effective Non-invasive	Low specificity: -False positives: tissues with rich micro vascularity (granulation tissue, inflammation, and oedema) -False negatives: regions with hyperkeratosis (Leukoplakia+++) or overgrowth of bacteria (producing extra fluorophores)	[5,72,73,74,75]
Targeted Fluorescence imaging (TFI)	Visualization of a fluorescence probe specifically targeting the neoplastic tissues	The targeted immune-fluorescence imaging: targeting an over-expressed protein by approved antibodies	Intra-tumour phenotype heterogeneity decreases it sensitivity	[5]
Narrow band imaging (NBI)	Visualization of the neoangiogenic patterns of tissues using an illumination light within the absorption spectrum of haemoglobin	The abnormal intra epithelial capillary loops (ICPL) patterns can be used to differentiate neoplastic from normal tissues The NBI endoscopic system is widely available	Characterization of IPCL patterns is subjective and false positive results are frequent (level of keratinization, lymphoid tissue, previous radiation or surgery, inflammation and vascular lesions)	[5,76,77]
High resolution microendoscopy (HRME)	Visualization of an emitted light by superficially applied fluorophores using a flexible fiber-optic probe placed in direct contact with the suspicious tissue	Cost effective Non-invasive High resolution High sensitivity and specificity Simple and portable device Requires minimal training High inter-rater reliability	Not commercially available The proflavine (the most commonly used contrast agent) is not approved for *in vivo* clinical use Limited field-of-view	[5,78]
Raman Spectroscopy (RS)	Visualization of the ‘molecular fingerprint’ (i.e., variations of chemical components) of a tissue using vibrational spectroscopic technique	Water absorption does not disturb the measurement High signal-to-noise ratio Fewer sample volumes are required for analysis.	Analyses are difficult No commercially available Too large for routine clinical use Time consuming	[5,79,80,81,82]

**Table 2:** Available cell lines to study oral premalignant disorders. (PMID: PubMed identification Member; ISSN: International Standard Serial Number).

Cell Line	Dysplasia Features	References
D9	Mild or Moderate dysplasia of ventral tongue	[48]
D20	Moderate dysplasia of lateral tongue	[48]
D34	Moderate dysplasia of posterolateral tongue	[48]
D38	Mild dysplasia of lateral tongue	[48]
DOK (dysplastic oral keratinocyte)	Epithelial dysplasia from the dorsal tongue of a 57-year-old heavy smoker	[89]
POE9n	Severely dysplastic oral epithelial lesion of a 65-year-old male possessing a homozygous deletion at the p16INK4A/p14ARF locus, lacking p53 expression, and exhibiting an extended but finite replicative life span	[90]
MSK Leuk1	Spontaneously derived from an oral leukoplakia lesion	[91]
Leuk1	Dysplastic leukoplakia adjacent an early invasive OSCC (T1N0M0) involving the tongue of a 47-year-old female	[92]
Leuk2	Dysplastic leukoplakia in a 72-year-old female with a history of recurrent new disease	[92]
LDOK	Severe dysplasia on the lingual alveolus, carrying a p53 gene mutation (G-T at codon 248) and does not express p16	[93]
CDOK	Mild dysplasia at the commissure	[93]
LTDOK	Mild dysplasia on the lateral tongue	[93]
SPDOK	Moderate dysplasia on the soft palate	[93]
VU-pre-SCC M3	Glottic laryngeal tumour with dysplasia in the mucosal resection margin	[47,94]
